# Molecular detection and phylogeny of *Ehrlichia canis* and *Anaplasma platys* in naturally infected dogs in Central and Northeast Thailand

**DOI:** 10.14202/vetworld.2022.2877-2889

**Published:** 2022-12-20

**Authors:** Andaman Purisarn, Sakulchit Wichianchot, Cherdsak Maneeruttanarungroj, Bandid Mangkit, Wuttinun Raksajit, Sarawan Kaewmongkol, Thitichai Jarudecha, Wanat Sricharern, Rucksak Rucksaken

**Affiliations:** 1 Department of Veterinary Technology, Faculty of Veterinary Technology, Kasetsart University, Bangkok, 10900, Thailand; 2 Department of Biology, School of Science, King Mongkut’s Institute of Technology Ladkrabang, Bangkok, 10520, Thailand; 3 Bioenergy Research Unit, School of Science, King Mongkut’s Institute of Technology Ladkrabang, Bangkok, 10520, Thailand

**Keywords:** *16S* rDNA gene, *Anaplasma platys*, *Ehrlichia canis*, *gltA* gene, phylogenetic analysis

## Abstract

**Background and Aim:**

*Ehrlichia canis* and *Anaplasma platys* are tick-borne, Gram-negative bacteria that cause canine monocytic ehrlichiosis and canine cyclic thrombocytopenia, respectively. These diseases are of great importance and are distributed globally. This study aimed to create new primers for the identification of *E. canis* and *A. platys* in naturally infected dogs using polymerase chain reaction (PCR), DNA sequencing, and phylogenetic analysis using the *16S* rDNA and *gltA* genes.

**Materials and Methods:**

In total, 120 blood samples were collected from dogs in three different locations (Saraburi, Buriram, and Nakhon Ratchasima provinces) in Central and Northeast Thailand. The molecular prevalence of *E. canis* and *A*. *platys* was assessed using PCR targeting the *16S* rDNA and *gltA* genes. All positive PCR amplicons were sequenced, and phylogenetic trees were constructed based on the maximum likelihood method.

**Results:**

*Ehrlichia canis* had an overall molecular prevalence of 15.8% based on the *16S* rDNA gene, compared to 8.3% based on the *gltA* gene. In addition, the overall molecular prevalence of *A. platys* using the *16S* rDNA gene was 10.8%, while the prevalence rate was 5.8% using the *gltA* gene. Coinfection was 0.8% in Saraburi province. The partial sequences of the *16S* rDNA and *gltA* genes of *E. canis* and *A. platys* in dogs in Central and Northeast Thailand showed 96.75%–100% identity to reference sequences in GenBank. Phylogenetic analysis of the *16S* rDNA and *gltA* genes revealed that *E. canis* and *A. platys* sequences were clearly grouped into their own clades.

**Conclusion:**

This study demonstrated the molecular prevalence of *E. canis* and *A. platys* in Central and Northeast Thailand. The *16S* rDNA and *gltA* genes were useful for the diagnosis of *E. canis* and *A. platys*. Based on the phylogenetic analysis, the partial sequences of the *16S* rDNA and *gltA* genes in *E. canis* and *A. platys* were related to prior Thai strains and those from other countries.

## Introduction

*Ehrlichia canis* and *Anaplasma platys* are Gram-negative, obligate, intracellular, tick-borne bacteria in the order Rickettsiales and family Anaplasmataceae [1, 2]. *Ehrlichia canis* and *A. platys* are important blood pathogens of dogs worldwide, especially in tropical and subtropical areas, and have recently been considered as zoonosis [3, 4]. *Ehrlichia canis* and *A. platys* are the causative agent of canine monocytic ehrlichiosis (CME) and canine infectious cyclic thrombocytopenia (CCT), respectively [5, 6]. The prevalence of *E. canis* in Thailand is in the range of 1.3%–38.3% [7–12], while the prevalence of *A. platys* in Thailand is in the range of 4.4%–30.6% [7–9, 11, 12]. Similar distributions of *E. canis* and *A. platys* have been reported in dogs in East and Southeast Asia [13, 14]. The CME can be characterized into three phases: Acute, subclinical (usually without clinical signs), and chronic. The clinical signs of a dog infected with *E. canis* may vary from asymptomatic to severe life-threatening disease [3, 14]. Symptoms of CME include depression, lethargy, high fever, anorexia, weight loss, pale mucous membranes, enlarged lymph nodes, petechiae caused by low platelets, anemia, bleeding, splenomegaly, hepatomegaly, lymphadenomegaly, and blindness [1, 15]. Cyclic thrombocytopenia caused by *A. platys* infection usually has mild or asymptomatic clinical signs [15, 16]. However, coinfections with *E. canis* can lead to severe thrombocytopenia [17].

The diagnosis of *E. canis* and *A. platys* infection can be performed based on blood smear examination under a 1000× light microscope. The morula stage of *E. canis* can be found in the monocytes and macrophages of infected dogs, whereas the morula of *A. platys* can be detected in the platelets of dogs [18, 19]. Although this method is easy to perform, it has low sensitivity, time-consuming, and requires experienced personnel to correctly identify the pathogens [9, 11, 17]. In addition, serological methods, such as indirect immunofluorescence assay and the enzyme-linked immunosorbent assay, have been widely used to diagnose *E. canis* and *A. platys* infection [6, 18]. These methods require specific equipment and may have specificity problems due to cross-reactions with other pathogens [17]. Polymerase chain reaction (PCR) is a highly sensitive and specific molecular method used for *E. canis* and *A. platys* detection [4, 20] and can be further used for phylogenetic analysis. Phylogenies are crucial tools for analyzing the evolutionary connections among different species or genes [21]. There have been recent reports on the use of *23S* rDNA [22], *16S* rDNA [22, 23], heat-shock operon (*groESL*) [22], and the *gltA* [22, 23] genes for phylogenetic analyses and characterization of *E. canis* and *A. platys* strains. The *16S* rRNA gene has been most commonly used for identifying *Ehrlichia* spp. [21]. Phylogenetic analysis of the *gltA* gene, the gene that encodes enzymes of the tricarboxylic acid cycle [24], exhibited higher variation among *Ehrlichia* and *Anaplasma* spp. [25, 26]. Therefore, *gltA* is one of the best genes for phylogenetic analysis of *Ehrlichia* species [26]. Previously, phylogenetic tree construction of *E. canis* and *A. platys* has been carried out in dogs based on the *16S* rDNA and *gltA* genes in many countries, such as the Philippines [27], Cuba [25], and China [28]. The *16S rDNA* and *gltA* nucleotide percentage identities vary from 99% to 100%. *E. canis* and *A. platys* are closely related genera that are commonly found coinfected [22]. However, there have been few reports on phylogenetic and epidemiological studies of *E. canis* and *A*. *platys* in Thailand, and no studies have been conducted in Saraburi, Buriram, and Nakhon Ratchasima provinces of Central and Northeast Thailand.

This study aimed to develop new primers for the detection of *E. canis* and *A. platys* in naturally infected dogs in Central and Northeast Thailand using PCR and to conduct phylogenetic analysis of *E. canis* and *A. platys* using the *16S* rDNA and *gltA* genes.

## Materials and Methods

### Ethical approval

This study was approved by the Animal Ethics Committee of the Faculty of Veterinary Technology, Kasetsart University, Bangkok, Thailand (ACKU62-VTN-0011).

### Study period and location

The samples were obtained from free-roaming, owned dogs from January 2021 to June 2022. The samples were collected from three different locations in Central and Northeast Thailand. The sample were processed at Kasetsart University, Bangkok, Thailand.

### Dog blood samples collection and genomic DNA extraction

**Figure-1:**
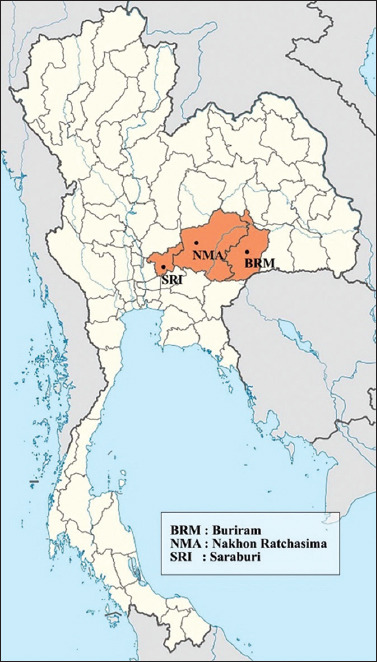
Map of sample collection sites highlighted in orange. [Source: Modified from https://commons.wikimedia.org/wiki/File:Thailand_location_map.svg].

The sample size was estimated using EpiTools (https://epitools.ausvet.com.au/) based on the previous prevalence [9]. To study both *E. canis* and *A. platys*, an estimated proportion of 0.044 has been used. The calculated sample size was 112 samples. In this study, 120 dog blood samples were obtained for better representation and to prevent data loss. Blood samples were collected from three different locations in Central and Northeast Thailand from Saraburi (n = 50), Buriram (n = 36), and Nakhon Ratchasima (n = 34) provinces (Figure-1). Blood samples (2 mL) were collected from the cephalic vein or saphenous vein and kept in ethylenediaminetetraacetic acid (EDTA) tubes, stored at −20°C until used. Blood smears were prepared on the day of collection. Positive control was made up of a sample that was positive for *E. canis* in a blood smear and confirmed using PCR as described by Wichianchot *et al*. [29]. Sterile distilled water was used as a negative control. Genomic DNA was extracted from 300 mL of each EDTA blood sample using a Genomic DNA Mini Kit (Geneaid^®^, New Taipei, Taiwan), according to the manufacturer’s instructions. The DNA was stored at −20°C until use and the DNA concentration was assessed using a Nanodrop Spectrophotometer (Thermo Fisher Scientific, Waltham, MA, USA).

### Primer design

The Primer3 software (https://bioinfo.ut.ee/primer3-0.4.0/) was used to create new PCR primers. The *16S* rDNA sequences of *E. canis* (GenBank accession no. GU810149.1) and *A. platys* (GenBank accession no. MK121782.1) and the *gltA* gene sequences of *E. canis* (GenBank accession no. AF304143.1) and *A. platys* (GenBank accession no. EU516387.1) were retrieved from the NCBI nucleotide database and used as DNA templates. The sequences for oligonucleotide primers and their predicted annealing temperatures as well as PCR product sizes are presented in Table-1.

**Table-1:** List of new primers used for the detection of *E. canis* and *A. platys* in this study.

Pathogen	Target gene	Primer name	Sequence (5’- >3’)	Annealing temperature (°C)	Product size (bp)
*E. canis*	*16S* rDNA	E16SeqF E16SeqR	TGCATGAGTCCAAGCCATA TAAGGTCCAGCCGAACTGA	57	1043 bp
	*gltA*	EglF EglR	ATGCCTCCTGAAATGGTTTG CCATCTCATACCACTGAGCA	54	847 bp
*A. platys*	*16S* rDNA	A16SeqF A16SeqR	GTGGCAGACGGGTGAGTAAT CTCATCGTTTACAGCGTGGA	54	683 bp
	*gltA*	AglF AglR	CGCCTGCAACTATCGAATG AGCGGTAGCAGAACTCAACG	58	1379 bp

*E. canis*=*Ehrlichia canis*, *A. platys*=*Anaplasma platys*

### Amplification of *E. canis* and *A. platys* of *16S* rDNA and *gltA* genes

Polymerase chain reaction (PCR) was performed to detect the *16S* rDNA and the *gltA* genes of *E. canis* and *A. platys*. All PCR reactions were prepared in 50 µL total volume in a 0.2 mL PCR tube, with each reaction containing 1X DreamTaq Green buffer (Thermo Scientific), 0.2 mM dNTP, 1 µM PCR primer, 1.25 units of DreamTaq DNA polymerase (Thermo Scientific), and 2 µL of DNA sample. The PCR conditions were as follows: All amplifications involved initial denaturation at 95°C for 2 min, 40 cycles of denaturation at 95°C for 30 s, annealing at 57°C for 30 s (for *E. canis 16S* rDNA), 54°C for 30 s (for *E. canis gltA* and *A. platys* *16S* rDNA), or 58°C for 30 s (for *A. platys gltA*) followed by the extension step at 72°C for 30 s. Then, all amplifications were completed with a final extension at 72°C for 5 min. The PCR products were resolved on a 2% agarose gel with DNA gel stain to identify predicted amplicons using the gel documentation system. All positive PCR amplicons were purified using a commercial gel extraction kit (Geneaid^®^) according to the manufacturer’s instructions and quantified using a Nanodrop spectrophotometry. Polymerase chain reaction products were sequenced from both ends using the Sanger method with a 3730XL automatic sequencer (Applied Biosystems, Foster City, CA, USA).

### Sequence analysis and phylogenetic tree construction

Nucleotide sequences were analyzed on the basis of the BlastN suite, available from the National Center for Biotechnology Information website (https://blast.ncbi.nlm.nih.gov/Blast.cgi). The similarity of nucleotide sequences was compared to the highest score hit. Phylogenetic trees were performed using the maximum likelihood (ML) method and Tamura-Nei model using the MEGA software version 10 (https://www.megasoftware.net/). A bootstrap of 1000 replicates was used for the reliability of the trees. A total of 49 positive samples were subjected to phylogenetic analysis. For the *16S* rDNA gene of *E. canis*, 9, 6, and 4 samples from the provinces of Saraburi, Buriram, and Nakhon Ratchasima, respectively, were used, while 4, 4, and 2 samples from Saraburi, Buriram, and Nakhon Ratchasima, respectively, were utilized for the *gltA* gene of *E. canis*. For *A. platys 16S* rDNA gene, there were 11, 1, and 1 samples from the provinces of Saraburi, Buriram, and Nakhon Ratchasima, respectively. For *A. platys gltA* gene, there were 6 and 1 samples from Saraburi and Buriram, respectively, and 57 reference sequences were retrieved from the NCBI database. Sequences from *Ehrlichia muris* (Accession number AB013009), *Ehrlichia chaffeensis* (Accession number MZ433238), *Ehrlichia ewingii* (Accession number M73227), *Ehrlichia ruminantium* (Accession number DQ647615), and *Rickettsia rickettsii* (Accession number DQ150694) were used as an outgroup for *E. canis* *16S* rDNA. Sequences from *E. muris* (Accession number MN685601), *E. chaffeensis* (Accession number AF304142), *E. ewingii* (Accession number DQ365879), *E. ruminantium* (Accession number DQ513397), and *R. rickettsii* (Accession number MT958042) were used as an outgroup for *gltA*. Sequences from *Anaplasma phagocytophilum* (Accession number DQ458805), *Anaplasma ovis* (Accession number AJ633052), *Anaplasma marginale* (Accession number AJ633048), *E. ewingii* (Accession number NR044747), and *R. rickettsii* (Accession number DQ150694) were used as an outgroup for *A. platys* *16S* rDNA. Sequences from *A. phagocytophilum* (Accession number JQ622145), *A. ovis* (Accession number KX579068), *A. marginale* (Accession number AF304140), and *R. rickettsii* (Accession number DQ150694) were used as an outgroup for *A. platys* *gltA*.

### Statistical analysis

Univariable analysis was performed using McNemar’s Chi-squared test for prevalence comparison between the *16S* rDNA and *gltA* genes. The statistical analysis was performed using the STATA software package version 15.1 (Stata Corporation, College Station, TX, USA). Results were considered significantly different for p < 0.05.

## Results

### Molecular prevalence of *E. canis* and *A. platys* infection

In total, 120 dog blood samples collected from Saraburi, Buriram, and Nakhon Ratchasima provinces were identified using PCR targeting of the *16S* rDNA and *gltA* genes of *E. canis* and *A. platys*. Physical examination revealed that no dog had signs of blood parasite infection. The overall prevalence of *E. canis* using the *16S* rDNA gene was 15.8%, the prevalence was 18% (9/50), 16.7% (6/36), and 11.8% (4/34) in Saraburi, Buriram, and Nakhon Ratchasima provinces, respectively. Using the *gltA* gene, the overall prevalence of *E. canis* was 8.3%, while the prevalence in each province was 8% (4/50), 11.1% (4/36), and 5.9% (2/34) in Saraburi, Buriram, and Nakhon Ratchasima provinces, respectively. Using the *16S* rDNA gene, the prevalence of *A. platys* was 10.8% overall and 22% (11/50), 2.8% (1/36), and 2.9% (1/34) in Saraburi, Buriram, and Nakhon Ratchasima provinces, respectively. In addition, the overall prevalence of *A. platys* using the *gltA* gene was 5.8%, and the prevalence in each province was 12% (6/50), 2.8% (1/36), and 0% (0/34) for Saraburi, Buriram, and Nakhon Ratchasima provinces, respectively (Table-2). The prevalence of the *16S* rDNA and *gltA* genes of *E. canis* was significantly different (p = 0.0027). In addition, the *16S* rDNA gene revealed coinfection in the Saraburi sample at a rate of 0.8% (1/120).

**Table-2:** PCR assay for the detection of *E. canis* and *A. platys* targeting *16S* rDNA and *gltA* genes in Saraburi, Buriram, and Nakhon Ratchasima provinces.

Province	No. of samples	No. of *E. canis* positive (%)		p-value^a^	No. of *A. platys* positive (%)		p-value^a^
	
		*16S* rDNA	*gltA*		*16S* rDNA	*gltA*	
Saraburi	50	9 (18.0%)	4 (8%)	0.0253	11 (22.0%)	6 (12.0%)	0.0588
Buriram	36	6 (16.7%)	4 (11.1%)	0.1573	1 (2.8%)	1 (2.8%)	1.0000
Nakhon Ratchasima	34	4 (11.8%)	2 (5.9%)	0.1573	1 (2.9%)	0 (0%)	0.3173
Total	120	19 (15.8%)	10 (8.3%)	0.0027	13 (10.8%)	7 (5.8%)	0.0578

a p-value from McNemar’s Chi-squared test, where p *≤* 0.05 was considered statistically significant. PCR=Polymerase chain reaction, *E. canis*=*Ehrlichia canis*, *A. platys*=*Anaplasma platys*

### Phylogenetic analysis

A total of 19, 10, 13, and 7 positive sequences were used for phylogenetic tree construction based on the ML method for *E. canis* *16S* rDNA, *E. canis* *gltA, A. platys* *16S* rDNA, and *A. platys* *gltA*, respectively.

**Figure-2:**
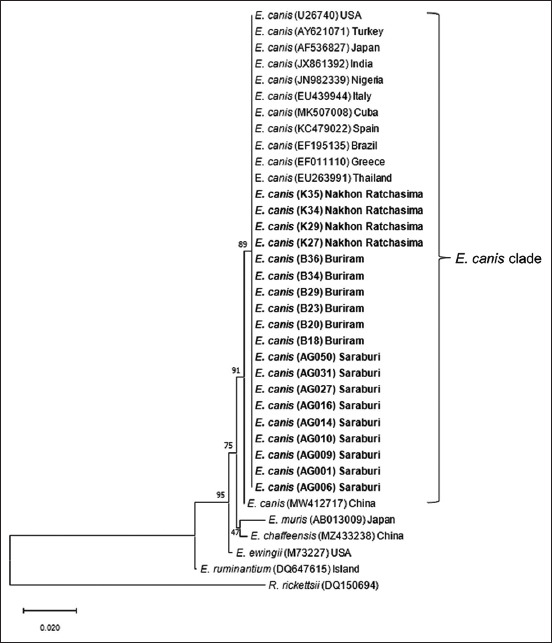
Phylogenetic analysis of *E. canis* based on nucleotide sequences of *16S* rDNA gene. The phylogenetic tree was constructed using the maximum likelihood method and bootstrap values were calculated based on 1000 replicates. Samples identified in this study are in bold. *E. canis=Ehrlichia canis*.

**Figure-3:**
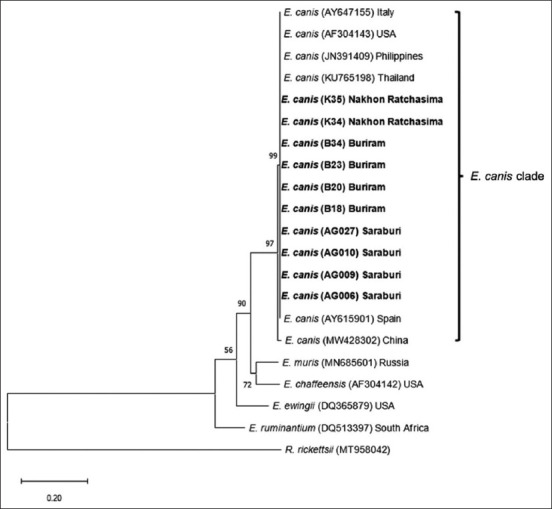
Phylogenetic analysis of *E. canis* based on nucleotide sequences of *gltA* gene. The phylogenetic tree was constructed using the maximum likelihood method and bootstrap values were calculated based on 1000 replicates. Samples identified in this study are in bold. *E. canis=Ehrlichia canis*.

The data indicated that sequences of *E. canis* *16S* rDNA in this study were closely related to other *E. canis* sequences obtained from the USA, Turkey, Japan, India, Nigeria, Italy, Cuba, Spain, Brazil, Greece, Thailand, and China (GenBank accession numbers U26740, AY621071, AF536827, JX861392, JN982339, EU439944, MK507008, KC479022, EF195135, EF011110, EU263991, and MW412717, respectively) (Figure-2) with the percentage of identity ranged from 99.59% to 100% (Table-3). The tree also showed that all *E. canis* sequences in this study were clustered in one clade, separated from *E. muris*, *E. chaffeensis*, *E. ewingii*, *E. ruminantium*, and *R. rickettsii*. The phylogenetic tree derived from the *gltA* gene showed that *E. canis* sequences in this study were clustered into one clade with other *E. canis* sequences from Italy, the USA, the Philippines, Thailand, Spain, and China (GenBank accession numbers AY647155, AF304143, JN391409, KU765198, AY615901, and MW428302, respectively) (Figure-3) with the percentage of identity ranged from 98.41% to 100% (Table-4). All *E. canis* sequences in this study were clustered in one clade, separated from *E. muris*, *E. chaffeensis*, and *E. ewingii*, *E. ruminantium*, and *R. rickettsii*.

**Table-3:** Pairwise identity (%) of *E. canis* *16S* rDNA sequences between Thai isolates in this study and global isolates. Percent identity among *E. canis* ranged from 99.59% to 100%.

Sample code	1	2	3	4	5	6	7	8	9	10	11	12	13	14	15	16	17	18
1. *R. rickettsii* DQ150694	100																	
2. *E. ruminantium* DQ647615 South Africa	85.43	100																
3. *E. muris* AB013009 Japan	84.5	97.57	100															
4. *E. chaffeensis* MZ433238 China	84.64	97.98	98.79	100														
5. *E. ewingii* M73227 USA	84.91	98.52	98.79	99.19	100													
6. *E. canis* MW412717 China	84.59	98.11	98.65	99.32	99.32	100												
7. All *E. canis* in this study^*^	84.5	97.84	98.38	99.06	99.06	99.73	100											
8. *E. canis* EU263991 Thailand	84.5	97.84	98.38	99.06	99.06	99.73	100	100										
9. *E. canis* EF011110 Greece	84.5	97.84	98.38	99.06	99.06	99.73	100	100	100									
10. *E. canis* EF195135 Brazil	84.5	97.84	98.38	99.06	99.06	99.73	100	100	100	100								
11. *E. canis* KC479022 Spain	84.5	97.84	98.38	99.06	99.06	99.73	100	100	100	100	100							
12. *E. canis* MK507008 Cuba	84.5	97.84	98.38	99.06	99.06	99.73	100	100	100	100	100	100						
13. *E. canis* EU439944 Italy	84.5	97.84	98.38	99.06	99.06	99.73	100	100	100	100	100	100	100					
14. *E. canis* JN982339 Nigeria	84.5	97.84	98.38	99.06	99.06	99.73	100	100	100	100	100	100	100	100				
15. *E. canis* JX861392 India	84.5	97.84	98.38	99.06	99.06	99.73	100	100	100	100	100	100	100	100	100			
16. *E. canis* AF536827 Japan	84.5	97.84	98.38	99.06	99.06	99.73	100	100	100	100	100	100	100	100	100	100		
17. *E. canis* AY621071 Turkey	84.5	97.84	98.38	99.06	99.06	99.73	100	100	100	100	100	100	100	100	100	100	100	
18. *E. canis* U26740 USA	84.37	97.71	98.25	98.92	98.92	99.59	99.87	99.87	99.87	99.87	99.87	99.87	99.87	99.87	99.87	99.87	99.87	100

* All samples including nine samples from Saraburi were AG001, AG006, AG009, AG010, AG014, AG016, AG027, AG031, and AG050, six samples from Buriram were B18, B20, B23, B29, B34, and B36, four samples from Nakhon Ratchasima were K27, K29, K34, and K35. *E. canis*=*Ehrlichia canis, R. rickettsii*=*Rickettsia rickettsii,* *E. ruminantium*=*Ehrlichia ruminantium, E. ewingii*=*Ehrlichia ewingii, E. canis*=*Ehrlichia canis, E. muris*=*Ehrlichia muris, E. chaffeensis*=*Ehrlichia chaffeensis*

**Table-4:** Pairwise identity (%) of *E. canis* *gltA* sequences between Thai isolates in this study and global isolates. Percent identity among *E. canis* ranged from 98.41% to 100%.

Sample code	1	2	3	4	5	6	7	8	9	10	11	12	13	14	15	16	17	18	19	20	21
1. *R. rickettsii* MT958042	100																				
2. *E. ruminantium* DQ513397 South Africa	52.5	100																			
3. *E. ewingii* DQ365879 USA	50.9	79.88	100																		
4. *E. canis* AG006 Saraburi	51.46	79.83	83.19	100																	
5. *E. canis* AG009 Saraburi	51.46	79.83	83.19	100	100																
6. *E. canis* AG010 Saraburi	51.46	79.83	83.19	100	100	100															
7. *E. canis* AG027 Saraburi	51.46	79.83	83.19	100	100	100	100														
8. *E. canis* B18 Buriram	51.46	79.83	83.19	100	100	100	100	100													
9. *E. canis* B20 Buriram	51.46	79.83	83.19	100	100	100	100	100	100												
10. *E. canis* B23 Buriram	51.46	79.83	83.19	100	100	100	100	100	100	100											
11. *E. canis* B34 Buriram	51.46	79.83	83.19	100	100	100	100	100	100	100	100										
12. *E. canis* K34 Nakhon Ratchasima	51.46	79.83	83.19	100	100	100	100	100	100	100	100	100									
13. *E. canis* K35 Nakhon Ratchasima	51.46	79.83	83.19	100	100	100	100	100	100	100	100	100	100								
14. *E. canis* KU765198 Thailand	51.46	79.83	83.19	100	100	100	100	100	100	100	100	100	100	100							
15. *E. canis* JN391409 Philippines	51.46	79.83	83.19	100	100	100	100	100	100	100	100	100	100	100	100						
16. *E. canis* AY647155 Italy	51.46	79.83	83.19	100	100	100	100	100	100	100	100	100	100	100	100	100					
17. *E. canis* AF304143 USA	51.46	79.83	83.19	100	100	100	100	100	100	100	100	100	100	100	100	100	100				
18. *E. canis* AY615901 Spain	51.46	79.83	83.19	100	100	100	100	100	100	100	100	100	100	100	100	100	100	100			
19. *E. canis* MW428302 China	51.03	79.16	82.56	98.41	98.41	98.41	98.41	98.41	98.41	98.41	98.41	98.41	98.41	98.41	98.41	98.41	98.41	98.41	100		
20. *E. muris* MN685601 Russia	50.57	79.95	83.43	85.8	85.8	85.8	85.8	85.8	85.8	85.8	85.8	85.8	85.8	85.8	85.8	85.8	85.8	85.8	85.43	100	
21. *E. chaffeensis* AF304142 USA	50.06	78.73	82.95	85.68	85.68	85.68	85.68	85.68	85.68	85.68	85.68	85.68	85.68	85.68	85.68	85.68	85.68	85.68	85.8	87.62	100

*R. rickettsii*=*Rickettsia rickettsii, E. ruminantium*=*Ehrlichia ruminantium, E. ewingii*=*Ehrlichia ewingii, E. canis*=*Ehrlichia canis, E. muris*=*Ehrlichia muris,* *E. chaffeensis*=*Ehrlichia chaffeensis*

**Figure-4:**
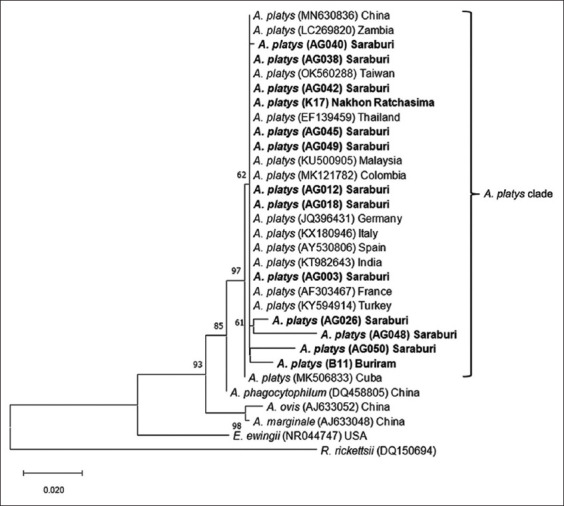
Phylogenetic analysis of *A. platys* based on nucleotide sequences of *16S* rDNA gene. The phylogenetic tree was constructed using the maximum likelihood method and bootstrap values were calculated based on 1000 replicates. Samples identified in this study are in bold. *A. platys*=*Anaplasma platys*.

**Figure-5:**
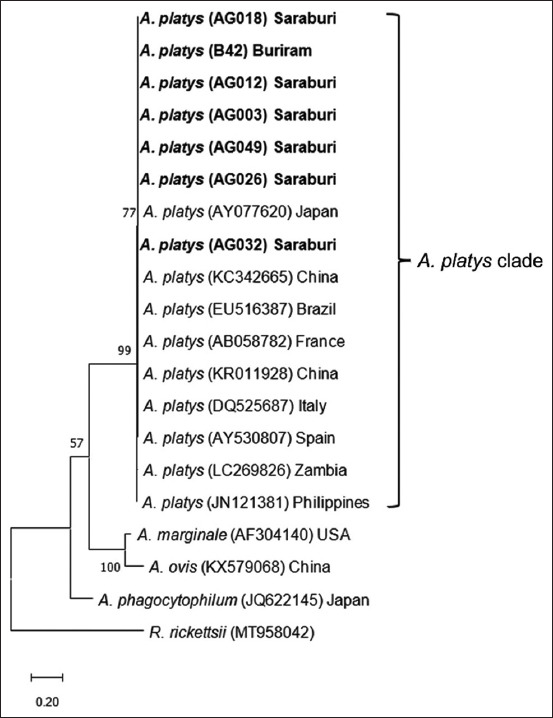
Phylogenetic analysis of *A. platys* based on nucleotide sequences of *gltA* gene. The phylogenetic tree was constructed using the maximum likelihood method and bootstrap values were calculated based on 1000 replicates. Samples identified in this study are in bold. *A. platys*=*Anaplasma platys*.

The *16S* rDNA tree showed that all *A. platys* sequences in this current research were clustered in one clade with other sequences from China, Zambia, Taiwan, Thailand, Malaysia, Colombia, Germany, Italy, Spain, India, France, Turkey, and Cuba (GenBank accession numbers MN630836, LC269820, OK560288, EF139459, KU500905, MK121782, JQ396431, KX180946, AY530806, KT982643, KY594914, AF303467, KY594914, and MK506833, respectively) (Figure-4) with the percentage of identity ranged from 96.75% to 100% (Table-5). The tree also showed that all these sequences of *A. platys* were separated from *A. phagocytophilum*, *A. ovis*, *A. marginale*, *E. ewingii*, and *R. rickettsii*. Similarly, the *gltA* tree showed that all *A. platys* in this study were clustered into one clade with other strains from Japan, China, Brazil, France, China, Italy, Spain, Zambia, and the Philippines (GenBank accession numbers AY077620, KC342665, EU516387, AB058782, KR011928, DQ525687, AY530807, LC269826, and JN121381, respectively) (Figure-5) with the percentage of identity ranged from 98.94% to 100% (Table-6). Furthermore, this tree showed that all the sequences of *A. platys* based on the *gltA* gene were separated from *A. marginale*, *A. ovis*, *A. phagocytophilum*, and *R. rickettsii*. All the new DNA sequences from this study were deposited in GenBank under accession numbers OP164592-OP164610 (*E. canis 16S* rDNA), OP164846-OP164858 (*A. platys 16S* rDNA), and OP270630-OP270646 (*E. canis gltA* and *A. platys gltA*)

**Table-5:** Pairwise identity (%) of *A. platys* *16S* rDNA sequences between Thai isolates in this study and global isolates. Percent identity among *A. platys* ranged from 96.75% to 100%.

Sample code	1	2	3	4	5	6	7	8	9	10	11	12	13	14	15	16	17	18	19	20	21	22	23	24	25
1. *R. rickettsii* DQ150694	100																		
2. *E. ewingii* NR044747 USA	84.31	100																							
3. *A. ovis* AJ633052 China	84.46	93.23	100																						
4. *A. marginale* AJ633048 China	84.62	93.38	99.23	100																					
5. *A. platys* AG048 Saraburi	81.69	91.38	94.92	95.23	100																				
6. *A. platys* AG050 Saraburi	82.62	92	95.54	95.85	96.77	100																			
7. *A. platys* B11 Buriram	82.69	92.58	95.98	96.29	96.75	97.37	100																		
8. A. phagocytophilum DQ458805 China	84.46	94.15	97.38	97.85	97.08	97.69	98.15	100																	
9. *A. platys* AG026 Saraburi	83.08	92.92	96.46	96.77	97.54	98	98.3	98.62	100																
10. *A. platys* MK506833 Cuba	83.85	93.54	97.08	97.54	97.69	98.31	98.76	99.38	99.23	100															
11. 7 *A. platys* from Saraburi*	83.69	93.54	97.08	97.38	97.85	98.46	98.92	99.23	99.38	99.85	100														
12. *A. platys* K17 Nakhon Ratchasima	83.69	93.54	97.08	97.38	97.85	98.46	98.92	99.23	99.38	99.85	100	100													
13. *A. platys* EF139459 Thailand	83.69	93.54	97.08	97.38	97.85	98.46	98.92	99.23	99.38	99.85	100	100	100												
14. *A. platys* OK560288 Taiwan	83.69	93.54	97.08	97.38	97.85	98.46	98.92	99.23	99.38	99.85	100	100	100	100											
15. *A. platys* MN630836 China	83.69	93.54	97.08	97.38	97.85	98.46	98.92	99.23	99.38	99.85	100	100	100	100	100										
16. *A. platys* LC269820 Zambia	83.69	93.54	97.08	97.38	97.85	98.46	98.92	99.23	99.38	99.85	100	100	100	100	100	100									
17. *A. platys* KU500905 Malaysia	83.69	93.54	97.08	97.38	97.85	98.46	98.92	99.23	99.38	99.85	100	100	100	100	100	100	100								
18. *A. platys* AF303467 France	83.69	93.54	97.08	97.38	97.85	98.46	98.92	99.23	99.38	99.85	100	100	100	100	100	100	100	100							
19. *A. platys* MK121782 Colombia	83.69	93.54	97.08	97.38	97.85	98.46	98.92	99.23	99.38	99.85	100	100	100	100	100	100	100	100	100						
20. *A. platys* KT982643 India	83.69	93.54	97.08	97.38	97.85	98.46	98.92	99.23	99.38	99.85	100	100	100	100	100	100	100	100	100	100					
21. *A. platys* JQ396431 Germany	83.69	93.54	97.08	97.38	97.85	98.46	98.92	99.23	99.38	99.85	100	100	100	100	100	100	100	100	100	100	100				
22. *A. platys* KY594914 Turkey	83.69	93.54	97.08	97.38	97.85	98.46	98.92	99.23	99.38	99.85	100	100	100	100	100	100	100	100	100	100	100	100			
23. *A. platys* KX180946 Italy	83.69	93.54	97.08	97.38	97.85	98.46	98.92	99.23	99.38	99.85	100	100	100	100	100	100	100	100	100	100	100	100	100		
24. *A. platys* AY530806 Spain	83.69	93.54	97.08	97.38	97.85	98.46	98.92	99.23	99.38	99.85	100	100	100	100	100	100	100	100	100	100	100	100	100	100	
25. *A. platys* AG040 Saraburi	83.54	93.38	96.92	97.23	97.69	98.31	98.76	99.08	99.23	99.69	99.85	99.85	99.85	99.85	99.85	99.85	99.85	99.85	99.85	99.85	99.85	99.85	99.85	99.85	100

* Seven samples from Saraburi were AG003, AG012, AG018, AG038, AG042, AG045, and AG049. *R. rickettsii=Rickettsia rickettsii, E. ewingii=Ehrlichia ewingii,* *A. platys=Anaplasma platys, A. phagocytophilum=Anaplasma phagocytophilum, A. marginale=Anaplasma marginale, A. ovis=Anaplasma ovis, A. marginale=Anaplasma marginale*

**Table-6:** Pairwise identity (%) of *A. platys* *gltA* sequences between Thai isolates in this study and global isolates. Percent identity among *A. platys* ranged from 98.94% to 100%.

Sample code	1	2	3	4	5	6	7	8	9	10	11	12	13	14	15	16	17	18	19	20
1. *R. rickettsii* MT958042	100																			
2. *A. platys* LC269826 Zambia	49.83	100																		
3. *A. platys* JN121381 Philippines	50.78	99.16	100																	
4. *A. platys* AG003 Saraburi	51.25	98.94	99.29	100																
5. *A. platys* AG0012 Saraburi	51.25	98.94	99.29	100	100															
6. *A. platys* AG0012 Saraburi	51.25	98.94	99.29	100	100	100														
7. *A. platys* B42 Buriram	51.25	98.94	99.29	100	100	100	100													
8. *A. platys* AG00149 Saraburi	51.35	99.05	99.38	99.92	99.92	99.92	99.92	100												
9. *A. platys* EU516387 Brazil	51.25	99.05	99.38	99.61	99.61	99.61	99.61	99.69	100											
10. *A. platys* DQ525687 Italy	51.35	99.26	99.56	99.69	99.69	99.69	99.69	99.77	99.77	100										
11. *A. platys* AY530807 Spain	51.35	99.26	99.56	99.69	99.69	99.69	99.69	99.77	99.77	99.84	100									
12. *A. platys* AY077620 Japan	51.25	99.05	99.56	99.61	99.61	99.61	99.61	99.69	99.69	99.77	99.77	100								
13. *A. platys* AG0026 Saraburi	51.25	99.16	99.65	99.69	99.69	99.69	99.69	99.77	99.77	99.84	99.84	99.92	100							
14. *A. platys* AG0032 Saraburi	51.25	99.16	99.65	99.69	99.69	99.69	99.69	99.77	99.77	99.84	99.84	99.92	100	100						
15. *A. platys* KC342665 China	51.25	99.16	99.65	99.69	99.69	99.69	99.69	99.77	99.77	99.84	99.84	99.92	100	100	100					
16. *A. platys* AB058782 France	51.35	99.26	99.56	99.77	99.77	99.77	99.77	99.84	99.84	99.92	99.92	99.84	99.92	99.92	99.92	100				
17. *A. platys* KR011928 China	51.35	99.26	99.56	99.77	99.77	99.77	99.77	99.84	99.84	99.92	99.92	99.84	99.92	99.92	99.92	100	100			
18. A. phagocytophilum JQ622145 Japan	52.05	60.49	61.25	61.52	61.52	61.52	61.52	61.43	61.16	61.25	61.25	61.07	61.16	61.16	61.16	61.25	61.25	100		
19. A. marginale AF304140 USA	50.4	60.61	61.73	60.94	60.94	60.94	60.94	61.02	61.02	61.02	61.1	60.94	60.94	60.94	60.94	61.02	61.02	62.91	100	
20. A. ovis KX579068 China	47.83	59.29	59.27	59.15	59.15	59.15	59.15	59.27	59.4	59.4	59.4	59.4	59.27	59.27	59.27	59.4	59.4	60.9	85.64	100

*R. rickettsii*=*Rickettsia rickettsii, A. platys*=*Anaplasma platys, A. phagocytophilum*=*Anaplasma phagocytophilum, A. marginale*=*Anaplasma marginale, A. ovis*=*Anaplasma ovis*

## Discussion

In this study, we developed new primers for *E. canis* and *A. platys* detection using the *16S* rDNA and *gltA* genes and used both genes for phylogenetic analysis. The overall prevalence rates using the *16S* rDNA gene (15.8% for *E. canis* and 10.8% for *A. platys*) were greater than those determined solely using the *gltA* gene (8.3% for *E. canis* and 5.8% for *A. platys*). Although only the overall prevalence between the *16S* rDNA and *gltA* genes of *E. canis* differed significantly (p = 0.0027), we suggest that *16S* rDNA is the better option for identifying species for both pathogens due to the higher detection rate. Molecular identification of *E. canis* and *A. platys* was performed in dog samples from three different locations (Saraburi, Buriram, and Nakhon Ratchasima provinces) in Central and Northeast Thailand. In this study, the overall prevalence of *E. canis* using the *16S* rDNA (15.8%) was higher than previously reported in Khon Kaen (1.3%) province, Thailand [8], but lower than previously reported from several parts of Thailand, including Bangkok (38.3%) [7], Maha Sarakham (21.5%) [10], Kalasin (25%) [11], and Buriram (36.7%) [12] provinces. The overall molecular prevalence of *A. platys* based on the *16S* rDNA gene was 10.8%; however, this prevalence was lower than previously reported in Bangkok (13.9%) [7], Buriram (30.6%) [12], and Kalasin (29.4%) [11] provinces, Thailand. The fluctuation in the prevalence percentage was possibly due to the living environment of the dogs [30]. The coinfection rate of *E. canis* and *A. platys* (0.8%) in this study was lower than our previous study in Buriram (14.2%) [12] and Kalasin (11.8%) [11] provinces, Thailand. In addition, coinfection rates of *E. canis* and *A. platys* in this study were lower than reported in studies in Saint Kitts (19%) [6], Grenada (4.5%) [22], and Nicaragua (4.7%) [31]. All dogs in this study showed no clinical symptoms of blood pathogen infection but several of them tested positive for either *E. canis* (15.8%) or *A. platys* (10.8%) using *16S* rDNA detection. These dogs may act as potential sources of zoonotic infection because it appears that most infections are asymptomatic [16]. Thailand is in a tropical area where infections with rickettsial pathogens are common. The results showed that the prevalence of *E. canis* and *A. platys* in this study was comparable to other tropical countries such as Indonesia, Malaysia, and Philippines [30, 32], India [33], Argentina [34], and Brazil [35].

The partial sequences of the *16S* rDNA and *gltA* genes of *E. canis* and *A. platys* in dogs in Central and Northeast Thailand were above 99.59% (*16S* rDNA) and 98.41% (*gltA*) identity to genotypes in GenBank. The current phylogenetic analysis for the *16S* rDNA and *gltA* genes of *E. canis* and *A. platys* agreed with the taxonomic separation of members of the family Anaplasmataceae into the *Ehrlichia* and *Anaplasma* genera [16]. The phylogenetic analysis of *16S* rDNA and *gltA* genes demonstrated that *E. canis* sequences are clustered tightly in *E. canis* subclade, whereas *E. muris*, *E. chaffeensis*, *E. ewingii*, *E. ruminantium*, and *R. rickettsii* clustering into their own subclades (Figures-2 and 3). However, it should be noted that even though *E. canis* were grouped in one clade; we found a small variation among *E. canis 16S* (MW412717) and *gltA* (MW428302) from China. For this specific sample from China, 12 widespread base substitutions were found in the DNA alignment to other *E. canis gltA* sequences. Other studies also reported no heterogeneity among *E. canis* groups using the *16S* rDNA gene [36]. A previous study in Thailand also showed that *E. canis* strains were linked with multiple connected branches and found little genetic diversity, suggesting slow and homogeneous evolution [17].

*Anaplasma platys* *16S* rDNA phylogenetic trees revealed that all of *A. platys* sequences from this study were clustered in the same clade with sequences from other countries (Figures-4 and 5). However, we found a small variation among *A. platys 16S* rDNA sequences from Saraburi and Buriram (AG026, AG048, AG050, and B11) as subclades in the tree. Although another sequence sample from Cuba (MK506833) contained a base substitution to other *A. platys* *16S* rDNA sequences, a high similarity of 96.75%–100% was observed in *A. platys 16S* rDNA sequences in concordance with a recent study in Khon Kaen province, Thailand [20]. The same pattern was also observed in *A. platys gltA* sequences (98.94%–100% similarity) which were clearly grouped into one clade with other *A. platys gltA* sequences.

## Conclusion

Using new PCR primers targeting the *16S* rDNA and *gltA* genes, this study provided the first molecular prevalence and phylogeny of *E. canis* and *A. platys* in asymptomatic dogs from Saraburi, Buriram, and Nakhon Ratchasima provinces, Central and Northeast Thailand. Phylogenetic analysis of the *16S* rDNA and *gltA* genes showed that *E. canis* and *A. platys* in Thailand were highly related to sequences from other countries. Future investigations on the genetic diversity of *E. canis* and *A. platys* should be conducted in different regions of Thailand.

## Authors’ Contributions

AP: Conducted the study and drafted the manuscript. SW: Conducted the study. CM: Methodology and edited the manuscript. BM, WR, SK, TJ, and WS: Reviewed and edited the manuscript. RR: Supervision, conceptualization, and reviewed and edited the manuscript. All authors have read and approved the final manuscript.
